# Chinese herbal medicine treatment based on subgroup differentiation as adjunct therapy for Parkinson’s disease: study protocol of a pilot add-on, randomised, controlled, pragmatic clinical trial

**DOI:** 10.1186/s13020-022-00572-0

**Published:** 2022-01-24

**Authors:** Sam Chun Sum Yuen, Ka Kit Chua, Linda L. D. Zhong, Kam Wa Chan, Conrad Kwan Ho Chan, Kam Leung Chan, Zhixiu Lin, Vincent Mok, Alexander Y. Lau, Min Li

**Affiliations:** 1 Mr. & Mrs. Ko Chi-Ming Centre for Parkinson’s Disease Research, School of Chinese Medicine, Hong Kong Baptist University, No.7 Baptist University Road, Kowloon Tong, Hong Kong; 2 Department of Medicine, The University of Hong Kong, Kowloon Tong, Hong Kong; 3 Hong Kong Institute of Integrative Medicine, Faculty of Medicine, The Chinese University of Hong Kong, Kowloon Tong, Hong Kong; 4 School of Chinese Medicine, Faculty of Medicine, The Chinese University of Hong Kong, Kowloon Tong, Hong Kong; 5 Department of Medicine and Therapeutics, Faculty of Medicine, The Chinese University of Hong Kong, Kowloon Tong, Hong Kong; 6 Department of Medicine & Therapeutics, Prince of Wales Hospital, Shatin, NT Hong Kong

**Keywords:** Traditional Chinese medicine, Parkinson disease, Pragmatic Clinical Trial

## Abstract

**Background:**

Parkinson’s disease (PD) is a prevalent and debilitating condition. Conventional medications cannot control all symptoms and may inflict adverse effects. A survey reported that Chinese herbal medicine (CHM) is frequently sought. Existing CHM trials were contradictory and often of poor quality due to lack of methodological rigor. A national clinical guideline was drafted in China with diagnostic criteria and treatment strategy of Chinese medicine (CM) patterns subgroups of PD. The suggested CHM were found to exhibit neuroprotective effect in in vitro and in vivo studies. This trial aims to preliminarily assess the effect of CHM prescribed based on pattern differentiation on PD symptoms and patients’ quality of life, and evaluate the feasibility of the trial design for a future large-scale trial.

**Methods:**

This trial will be a pilot assessor- and data analyst blind, add-on, randomised, controlled, pragmatic clinical trial. 160 PD patients will be recruited and randomised into treatment or control groups in a 1:1 ratio. The trial will be conducted over 32 weeks. PD patients in the treatment group will be stratified into subgroups based on CM pattern and receive CHM accordingly in addition to conventional medication (ConM). The control group will receive ConM only. The primary outcome will be part II of the Movement Disorder Society Sponsored Revision of Unified Parkinson’s Disease Rating Scale (MDS-UPDRS). Secondary outcomes will include part and total scores of MDS-UPDRS, domain and total scores of Non-motor symptom scale (NMSS). Adverse events will be monitored by monthly follow-ups and questionnaires. Mixed models will be used to analyse data by Jamovi and R.

**Expected outcomes:**

The success of our trial will show that the pragmatic design with subgroup differentiation is feasible and can produce reliable results. It will also provide preliminary data of the effect of CHM on improving clinical outcomes and quality of PD patients. Data collected will be used to optimize study design of the future large-scale clinical study.

**Ethical clearance:**

Ethical clearance of this study was given by the Research Ethics Committee of Hong Kong Baptist University (REC/20-21/0206).

*Trial registration *This trial is registered on ClinicalTrials.gov (NCT05001217, Date: 8/10/2021, https://clinicaltrials.gov/ct2/show/NCT05001217). Type of manuscript: clinical trial protocol (date: 3^rd^ November, 2021, version 1)

**Supplementary Information:**

The online version contains supplementary material available at 10.1186/s13020-022-00572-0.

## Background

Parkinson’s disease (PD) is the second most common neurodegenerative disease in the world. There are more than 13,000 PD patients in Hong Kong, constituting approximately 0.5% in the age group of 55 years old or above [1]. Motor and non-motor symptoms (MS and NMS) are the two main categories of clinical presentations of PD that affect the quality of life of PD patients [2–4]. Conventional medicine (ConM), including medications such as Levodopa and dopamine agonists, is effective in improving motor function, but not postural and gait problems nor most NMS [5, 6]. ConM also incurs adverse effects, including dyskinesia and freezing of gait [7]. Thus, some patients seek complementary and alternative therapies (CATs) in the hope to improve their conditions.

A survey of PD patients in China revealed that more than half of them had sought CATs [8]. Another study in China showed that Chinese herbal medicine (CHM) and acupuncture, two commonly used Chinese medicine (CM) treatments, were the two most popular choices of CATs [9]. There have been numerous attempts to analyse the effectiveness of CHM through clinical trials. For instance, clinical studies showed that CHM was effective in improving five domains of the Parkinson's Disease Questionnaire (PDQ-39), including mobility and bodily discomfort, and improving NMS [10, 11]. However, a systematic review concluded that most clinical studies of CHM contained biases and methodological inadequacy, so that the effectiveness of CHM could not be estimated accurately [12]. Among them, the omission of CM pattern differentiation in the study design was highlighted as a major limitation in these trials.

Differentiation of CM patterns is an important diagnostic procedure that Chinese medicine practitioners (CMPs) prescribe CHM accordingly [13]. Observational studies reported that CM patterns were associated with PD subtypes, and responded differently to treatment. The Internal Stirring of Liver Yang and Wind, for instance, was related to tremor dominant Parkinson’s disease, and was most responsive to add-on CHM therapy when compared to other CM patterns [14, 15]. However, there is no existing CHM clinical trial on PD incorporated the CM pattern differentiation in the study design with satisfactory control on bias.

In 2008, a CM clinical guideline (the Guideline) for PD was issued by the Chinese government [16]. The Guideline, developed based on clinical evidence and expert consensus, included the diagnostic criteria and treatment strategy of CM patterns frequently observed among PD patients [16]. The CHM suggested by the Guideline were reported to exhibit neuroprotective effects in in vivo and in vitro studies. The CHM for the Internal stirring of Liver Yang and Wind, for example, was associated with a decreased MPP^+^-induced oxidative damage and lowered neuronal apoptosis in neuronal cultures, and reduced neuronal loss in substantia nigra pars compacta in PD mice models [17]. Other CHM formulae in the Guideline were associated with elevated dopamine, noradrenaline and 5-hydroxytryptamine levels, and decreased monoamine oxidase B in the striatum of mice models [18, 19]. However, no clinical trial was conducted to evaluate the effectiveness and feasibility of the Guideline on PD clinical outcomes. In light of this, our study aims to evaluate the feasibility and preliminarily assess the effectiveness of integrative medicine treatment combining ConM and CHM in improving clinical outcomes and quality of life of PD patients. The treatment strategy will be based on the Guideline.

## Methods

### Study design

**Fig. 1:**
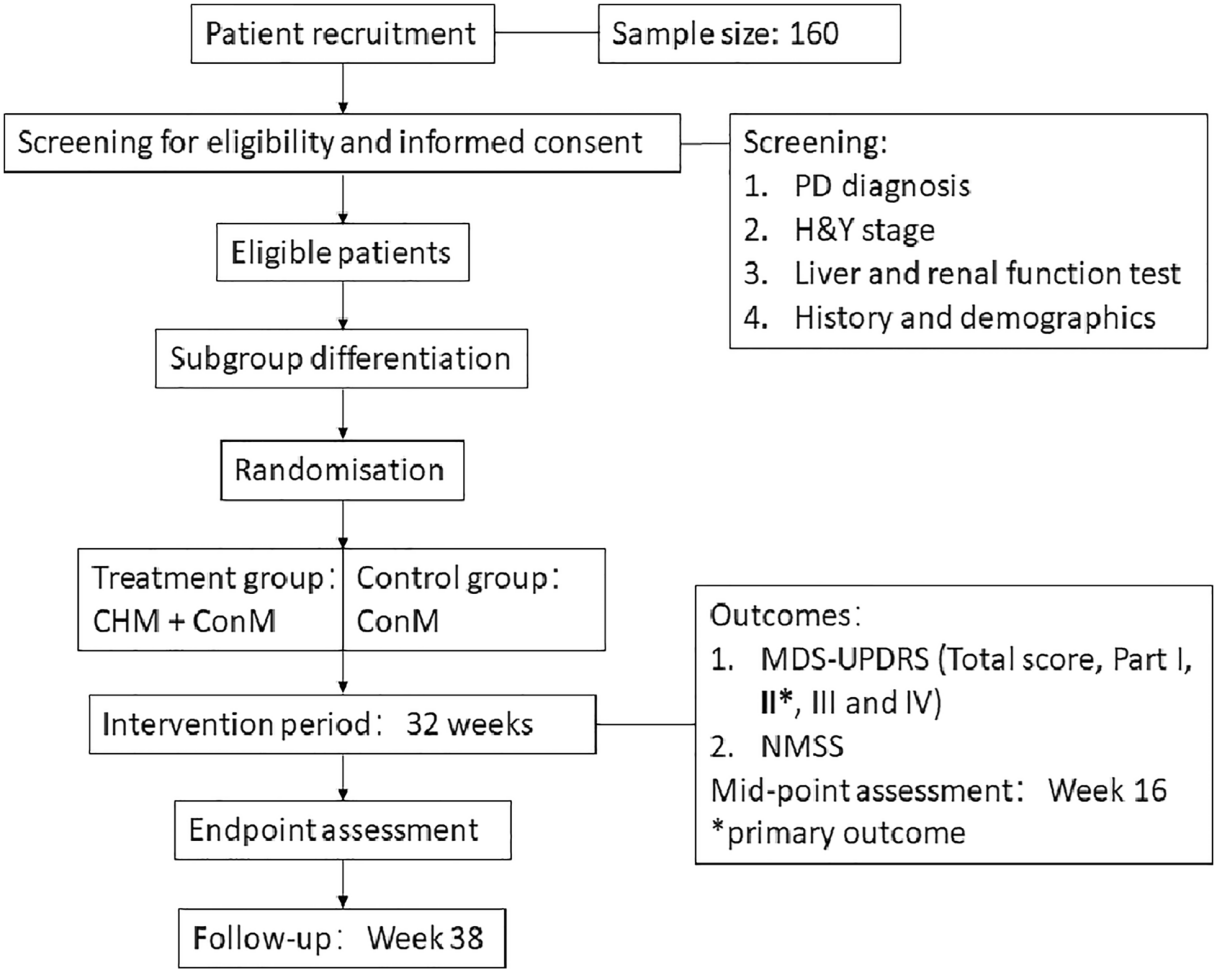
Study flowchart

This study will be an add-on, assessor- and data analyst-blind, randomised, controlled, pragmatic trial. This protocol adheres to the Standard Protocol Items: Recommendations for Interventional Trials (SPIRIT) statement [20] (Additional file 1). Participants in treatment group will be given integrated medicine treatment combining ConM and CHM. They will be stratified into subgroups based on their Chinese medicine patterns, and receive CHM accordingly. The control group will receive ConM only. The intervention period will be 32 weeks. The flow of the study is shown in Fig. 1. Table 1 shows the schedule of enrolment, interventions, and assessments. This study is registered on ClinicalTrials.gov (ref. no.: NCT05001217, Additional file 2).

**Table 1:** Schedule of enrolment, interventions, and assessments

Timepoint	Study period						
	Enrolment	Allocation	Post-allocation				Follow-up
	*-t*_*1*_	0	*t*_*1*_ (Week 1–4)	*t*_*2*_* (after 4 weeks)*	*t*_*3*_* (after 16 weeks)*	*t*_*4*_* (after 32 weeks)*	*t*_*5*_* (after 38 weeks)*
**Enrolment**:							
Eligibility screen	X						
Informed consent	X						
Medical history	X						
Renal and Liver function test	X			X		X	
Chinese medicine pattern differentiation	X						
Allocation		X					
**Interventions:**							
Chinese herbal medicine (for treatment group)			X	X	X	X	
Compliance to intervention (for treatment group)			X	X	X	X	
Conventional medication (for all groups)			X	X	X	X	
**Assessments:**							
MDS-UPDRS Part II		X			X	X	X
MDS-UPDRS Part I, III, IV, total scores		X			X	X	X
NMSS		X			X	X	X
**Adverse events**					X	X	

### Inclusion and exclusion criteria

Patients who are (1) diagnosed with PD according to United Kingdom Parkinson's Disease Society Brain Bank (UKPDBB) diagnostic criteria [21]; (2) aged 18 to 80 years old; and (3) under stable ConM treatment with no alteration of dosage in the past 30 days [22] will be included. Patients with one of the following conditions will be excluded: (1) secondary PD or atypical Parkinsonian disorder; (2) used antidepressants in the past 30 days; (3) with concurrent psychiatric, mood, or other neurological disorders; (4) suicidal (with suicidal thoughts in the past year); (5) with concurrent severe disorders, such as cancer or myocardial infarction; (6) participation in another CHM clinical study; (7) pregnant or breast-feeding; (8) Hoehn and Yahr (H&Y) stage 4 or above; (9) liver and renal function derangement; (10) does not speak Chinese or English; or (11) Hong Kong version of Montreal Cognitive Assessment 5-min protocol < 22 points [23].

### Recruitment and setting

Participants will be recruited from Hong Kong Parkinson’s Disease Association, clinics of the School of Chinese Medicine of Hong Kong Baptist University (HKBU), the Faculty of Medicine of The Chinese University of Hong Kong (CUHK) and the Queen Elizabeth Hospital (QEH). The mentioned sites are in contact of a substantial pool of PD patients. Posters, mail, and email will be the major means of advertisement. Patients interested in participating in the study will be referred to research assistant I (RA-I) for screening. The Hong Kong version of the Montreal Cognitive Assessment 5-min protocol (HK-MOCA 5 min-protocol) will be used to screen out PD patients susceptible to mild cognitive disorder (MCD) [23]. A score of 22 or less was suggested to be the cut-off for detecting patients susceptible to MCI that renders them unable to provide informed consent [24]. The details and responsibilities regarding participation in this study will be explained to eligible participants. Written consent will be obtained from each participant afterwards. All participants will be stratified into four subgroups based on CM patterns according to their clinical presentations. Each will then be randomly allocated to the treatment group or control group.

### Randomisation and blinding

Stratified block randomisation will be used in this study. The block size will be random, either 4 or 8. Participants will be stratified according to their H&Y stage and CM pattern subgroup (there will be 12 strata altogether). A random number sequence will be generated, encrypted and locked by research assistant II (RA-II) using a computer program, Random Allocation Software. A sealed opaque envelope will be used to keep the hard copies of the allocation sequence secure. Soft copies of the allocation results will be password-protected. The allocation will be concealed from personnel involved in the recruitment of participants, screening, CM subgroup differentiation, assessment and data analysis. Participants will be randomised into treatment and control groups in a ratio of 1:1.

Measures will be taken to ensure assessor blinding. Firstly, patients will be reminded to not reveal their group allocation to the assessors. Secondly, assessors will not have access to any notes or files containing patients’ data. If a patient’s allocation is revealed, the assessor will be replaced by another [25].

### Intervention

The intervention period will be 32 weeks. Participants in the treatment group will receive integrated medicine treatment combining CHM and ConM. Patients must display all presentations of one of the four patterns to be stratified into that pattern subgroup. The diagnostic criteria of the four CM subgroups are listed as follows:

1. The “Phlegm-Heat-stirring Wind” subgroup:
  The primary presentations are lassitude of spirit, lack of strength, chest tightness and bitter taste in the mouth.
2. The “Spleen- and Kidney-Yang Deficiency” subgroup:
  The primary presentations are fear of cold, shortness of breath, disinclination to talk, clear and large quantity of urine or occasional urinary incontinence.
3. The “Internal Stirring of Yang and Wind” subgroup:
  The primary presentations are dizziness or headache, dry mouth, irritability, and neck stiffness.
4. The “Qi Deficiency and Stasis of Blood” subgroup:
  The primary presentations are shortness of breath, lack of strength, stabbing pain in the body, and darkish complexion.

Each CM subgroup will receive a specific formula. The details are as follows:

1. The “Phlegm-Heat-stirring Wind” subgroup:
  Pinelliae Rhizoma (Jiang Ban Xia), Bambusae Caulis in Taenias (Zhu Ru), Aurantii Fructus Immaturus (Zhi Shi), Citri Reticulatae Pericarpium (Chen Pi), Zingiberis Rhizoma Recens (Sheng Jiang), Glycyrrhizae Radix et Rhizoma (Gan Cao), Poria (Fu Ling), Jujubae Fructus (Da Zao), Uncariae Ramulus cum Uncis (Gou Teng), Bombyx Batryticatus (Jiang Can), Coptidis Rhizoma (Huang Lian), Scutellariae Radix (Huang Qin)
2. The “Spleen- and Kidney-Yang” subgroup:
  Poria (Fu Ling), Moutan Cortex (Mu Dan Pi), Alismatis Rhizoma (Ze Xie), Rehmanniae Radix (Shu Di Huang), Corni Fructus (Shan Zhu Yu), Dioscoreae Rhizoma (Shan Yao), Aconiti Lateralis Radix Praeparata (Pao Fu Zi), Cinnamomi Cortex (Rou Gui), Morindae Officinalis Radix (Ba Ji Tian), Cistanches Herba (Rou Cong Rong), Testudinis Carapax et Plastrum (Gui Pan), Trionycis Carapax (Bie Jia)
3. The “Internal Stirring of Yang and Wind” subgroup:
  Rehmanniae Radix (Shu Di Huang), Corni Fructus (Shan Zhu Yu), Dioscoreae Rhizoma (Shan Yao), Poria (Fu Ling), Moutan Cortex (Mu Dan Pi), Alismatis Rhizoma(Ze Xie), Haliotidis Concha (Shi Jue Ming), Polygoni Multiflori Caulis (Ye Jiao Teng), Taxilli Herba (Sang Ji Sheng), Uncariae Ramulus cum Uncis (Gou Teng), Poria cocos (Schw.) Wolf (Fu Shen), Eucommiae Cortex (Du Zhong), Leonuri Herba (Yi Mu Cao), Achyranthis Bidentatae Radix (Niu Xi), Gardeniae Fructus (Zhi Zi), Scutellariae Radix (Huang Qin), Gastrodiae Rhizoma (Tian Ma)
4. " The “Qi Deficiency and Stasis of Blood” subgroup:
  Persicae Semen (Tao Ren), Carthami Flos (Hong Hua), Angelicae Sinensis Radix (Dang Gui), Chuanxiong Rhizoma (Chuang Song), Paeoniae Radix Alba (Bai Shao), Rehmanniae Radix (Shu Di Huang), Astragali Radix (Huang Qi), Angelicae Sinensis Radix (Dang Gui), Pheretima (Di Long), Pinelliae Rhizoma (Jiang Ban Xia), Bambusae Caulis in Taenias (Zhu Ru), Aurantii Fructus Immaturus (Zhi Shi), Citri Reticulatae Pericarpium (Chen Pi), Zingiberis Rhizoma Recens (Sheng Jiang), Glycyrrhizae Radix et Rhizoma (Gan Cao), Poria (Fu Ling), Jujubae Fructus (Da Zao), Polygalae Radix (Yuan Zhi), Acori Tatarinowii Rhizoma (Shi Cang Pu), Uncariae Ramulus cum Uncis (Gou Teng)

The diagnostic criteria of CM and the CHM treatment plans are based on the Guideline, which issued by the National Administration of Traditional Chinese Medicine in China. It is titled “*Guidelines for Diagnosis and Treatment of Common Internal Diseases in Chinese Medicine Diseases of Modern Medicine”* [26]. The Guideline was based on expert consensus and clinical evidence. In this study, the treatment protocol will, in addition, include minor adjustments of the CHM formula according to the presentations of individual patients. Registered CMPs will be responsible for the prescription and preparation of CHM. The CHM prescribed for each patient will compose of registered proprietary CHM and single CHM granules for prescription. Each patient’s granules will be prepared as individual packets. Participants will be instructed to prepare each packet for consumption by dissolving the contents in 200 ml boiling water, and to take the medication twice per day, 7 days per week, for 32 weeks. Participants in the control group will receive only ConM. The reporting of all mentioned CM terms adhere to the World Health Organization international standard terminologies on traditional medicine in the Western Pacific Region [27].

### Herbal safety

To ensure consistent quality of the CHM, the production of the CHM granules will adhere to the standards of Good Manufacturing Practice (GMP). Prescribing of the CHM will take place in clinics in the School of Chinese Medicine at HKBU. Follow-up consultations will be conducted monthly to monitor the condition of participants. During these consultations, a questionnaire will be given to patients for the report of adverse events. Severe adverse events will be reported immediately to RA-I via telephone. CHM with known toxicity listed in Schedule 1, Chapter 549, Chinese Herbal Medicines, Chinese Medicine Ordinance of Hong Kong will not be used. The liver and renal function of participants will be tested prior to the start of the study, one month after the start of intervention and at the end of the intervention period (32 weeks), in which patients’ blood will be extracted and analysed in a medical laboratory. Additionally, participants will be asked to keep a record of any days on which they did not take the CHM and to report the reason.

### Outcome assessment

Outcome assessment will be done at baseline, week 16 and week 32. The primary outcome will be the Movement Disorder Society Sponsored Revision of Unified Parkinson’s Disease Rating Scale (MDS-UPDRS) Part II. MDS-UPDRS is a study-validated comprehensive assessment of MS, NMS and quality of life of PD patients, combining self-completed questionnaires and investigator examination [28]. Part II of MDS-UPDRS assesses the self-reported motor experience during daily life of PD patients. Secondary outcomes include sub-total scores in Parts I, III and IV as well as the total score of MDS-UPDRS, and the domain and total score on the Non-motor symptom scale (NMSS). To observe the lingering effect of CHM after the cessation of treatment, a follow-up assessment will be conducted 6 weeks after the end of the intervention period. Baseline demographics, including sex, age, disease duration and severity, and ConM details, will be collected during baseline assessment.

To monitor the side effects of the integrated medicine treatment, patients will be asked to fill in a questionnaire on adverse events during each outcome assessment session after the study begins. The grading of severity of adverse events will be based on Common Terminology Criteria for Adverse Events (CTCAE) Version 5.0. In general, adverse events will be categorized into 5 grades:

1. Grade 1: mild, possibly asymptomatic, based on clinical or diagnostic observations only; intervention not indicated.
2. Grade 2: moderate; minimal, local or non-invasive intervention indicated; limiting age-appropriate instrumental activities of daily living.
3. Grade 3: severe or medically significant but not immediately life-threatening; hospitalization or prolongation of hospitalization indicated; disabling; limiting self-care activities of daily living.
4. Grade 4: life-threatening consequences; urgent intervention indicated.
5. Grade 5: death related to adverse events.

All assessors will be trained by the Principal Investigator (PI) before the commencement of the study. If an assessment session is needed to be canceled for any reason, it will be rescheduled within a week of the designated date.

### Sample size calculation

There are numerous methods for sample size calculation for pilot trials. It is suggested that 12–30 participants per arm will be sufficient [29]. However, to better generalize our findings, we have estimated the sample size of our study based on significance, power, estimated effect size and drop-out rate. As no study investigating the efficacy of CHM with MDS-UPDRS has been done, we used UPDRS scores as reference. According to a systematic review, the pooled effect of CHM, shown in Cohen’s d, was 0.69 (moderate effect) [30, 31]. Therefore, to test whether the treatment plan in this study achieves an effect size of 0.5 (expressed in Cohen’s d), with a power of 90% and a confidence of 95% (two-tailed), and assuming a drop-out rate of 15%, the sample size needed will be 160 in total. The calculation was done using G Power.

### Data analysis

The software Jamovi and R will be used to analyse data. The intention-to-treat approach will be adopted. The change in the primary outcome and secondary outcome between baseline and the end of the intervention period (week 32) will be evaluated. Regression models will be used to evaluate the difference of change in outcome between treatment and control groups. Subgroup analysis will also be conducted comparing the outcome of each CM subgroup in treatment the matching subgroup in the control group, and between H&Y stages. Missing values will be accounted for by regression analysis.

Continuous variables following normal distribution will be expressed in means and 95% confidence intervals. Non-parametric data will be expressed in median and interquartile ranges. Categorical variables will be expressed in percentages and confidence intervals. For primary and secondary outcomes, the adjusted means, standard deviation, 95% confidence interval, and effect size will be presented. Adverse events will be presented as the number of cases and proportions, which will be compared between treatment and control group. Research assistant III (RA-III), who will be blinded to group allocation results, will be responsible for data analysis.

Sensitivity analyses will be done on missing data (missing cases imputed with regression and imputed with last-observation-carried-forward), and non-compliance (per-protocol analysis). Per protocol is defined as included cases that highly adhered to the intervention programme (two cut-offs of CHM compliance, 60% and 80%, will be used for analysis) and completed all outcome assessments.

### Interim data analysis

Interim data analysis is planned when 50% of the designated sample size completed the mid-point assessment (week 16) of outcome assessment. The purpose is early detection of serious or unanticipated adverse events, and analysis of effectiveness. Detection of harm induced by the treatment plan will lead to the early cessation of this study [32].

### Data management

Hardcopies of the data will be kept in locked safes with limited access. All data will be entered electronically. Participants will be assigned code numbers as identifiers. Documents, including assessment forms and reports, will be identified using the code number to maintain confidentiality. A hard disk drive, which will be kept by RA-II, will be used to back up data. The trial management committee will be formed by research team members. RA-III will be responsible for managing and sending a copy of the dataset to the committee regularly. The committee will hold meetings monthly to update each other on progress and to discuss any issues that may arise. Data will be entered twice and cleaned before analysis. Data storage and transfer will be secured with a password to ensure data privacy.

### Compliance strategy and withdrawal management

The compliance management program is as follows. Firstly, participants will be instructed to return all unconsumed CHM in each follow-up, so that their monthly CHM dosage consumption can be calculated. Second, the email and phone number of the contact person, RA-I, will be given to participants, so as to enhance two-way communication. Reminders and details regarding the study will be sent to participants via telephone, instant message and email, while patients will be encouraged to make inquiries and express their concerns via the said means of communication. Third, compensation will be given to those who complete the entire study. If any participant wishes to withdraw, our research team will seek to understand his or her concern and to resolve the problem. Patients who decide to withdraw, especially for the controls, will be encouraged to at least complete self-reported online assessments, so that not all data will be missing. To ensure that participants adhere to the treatment plan, which is essential for guaranteeing treatment quality, a journal will be given to patients to keep a record of any days on which they did not take their CHM and the reason for not doing so. They will also be asked to bring all unconsumed CHM to their next monthly visit, so that their CHM compliance can be calculated.

### Termination criteria

A patient’s participation will be terminated prematurely if he or she experiences any of the following: (1) severe adverse effects or allergy after CHM treatment; (2) pregnancy; or (3) participation in other clinical studies. An adverse event meeting one or more of the following criteria will be defined as serious: The event results in death, is life-threatening, causes hospitalisation or prolongs hospitalisation, incurs disability, or leads to an event that requires immediate intervention to prevent the mentioned conditions from taking place. The entire study will be terminated if (1) there is the occurrence of any serious adverse event(s) related to the consumption of CHM, or (2) completion of all follow-up assessments.

## Discussion

Despite efforts to evaluate the efficacy of CHM in treating Parkinson’s disease, the clinical studies fail to meet the rigorous standards needed to ensure reliable, accurate results. Moreover, these studies, adopting the traditional explanatory design, failed to differentiate patients based on CM patterns, and hence assign treatment that match his or her particular condition. This is considered to be a fundamental study design flaw. A pragmatic trial design, which is used to evaluate complex interventions administered in realistic clinical conditions, may be a better alternative [33]. The purpose of a pragmatic trial is to assess a general treatment approach rather than a specific drug; therefore, adjustment of treatment, including the type of drug and dosage, is usually allowed, such that the treatment reflects actual clinical practice. Thus, by adopting the pragmatic design, CM pattern differentiation can be incorporated into a clinical study.

While there are concerns that the pragmatic design will undermine study rigor, a recent systematic review showed that an increase in pragmatism in CM trials, with parameters including flexibility in intervention delivery, the strictness of eligibility and adherence to the clinical setting, was not related to more bias [34]. Indeed, the pragmatic design has not only been used to study CHM, but also in drug trials. A study, seeking to compare the effectiveness of levodopa, dopamine agonist and monoamine oxidase type B inhibitors for newly diagnosed PD patients, adhered to actual clinical practice to derive real-world evidence [35]. Clinicians were free to choose any medication within the assigned drug class and titrate dosage according to the condition of individual patients. Despite its pragmatism, the rigor of the study was not compromised. In addition, there are validated tools and guidelines to standardize the methodology of pragmatic trials, such as the PRECIS-2 tool, the CONSORT extension for pragmatic trials, and the guideline for developing and evaluating complex intervention by the MRC and NIHR [32, 36, 37].

One potential weakness is that our study will be an open-label trial without a placebo as control. This would possibly incur confounding bias and not be able to nullify the placebo effect. As our trial aims to evaluate the actual clinical practice of CM, the CMPs of our study will need to assess a patient’s condition before prescribing a personalized CHM for him/her, making blinding and placebo unfeasible. To lower assessment bias, assessors will be blinded. The assessor will be swapped when the allocation of a patient is revealed.

Another limitation of this study is the sole focus on clinical outcomes. Including objective parameters as assessments, such as biomarkers, would increase objectivity and reduce assessment bias. However, considering patient-reported outcomes are often omitted in previous trials, and of more interest to patients, our trial opted to focus on such outcomes.

## Conclusion

The success of our trial will have three major impacts. First, our trial will show that a rigorous CHM pragmatic trial is feasible and provides reliable results. Second, there will be preliminary evidence indicating that CHM, prescribed according to CM pattern differentiation, can improve clinical outcomes and quality of life for PD patients. Third, our trial will preliminarily indicate that CM pattern differentiation, with CHM prescribed accordingly, is an effective clinical practice. Data obtained in this study will be used to optimise study methodology in the future large-scale pragmatic.

## Supplementary Information

**Additional file 1.** SPIRIT checklist (it is a document providing evidence-based recommendation of the content and format of a clinical trial protocol).

**Additional file 2.** World Health Organization Trial Registration Data Set (this document outlines the basic information of this trial as registered on the clinical trial registry).

## Data Availability

Not applicable.

## Abbreviations

PD: Parkinson’s disease
MS: Motor symptoms
NMS: Non-motor symptoms
ConM: Conventional medicine
CATs: Complementary and alternative therapies
CHM: Chinese herbal medicine
CM: Chinese medicine
PDQ: Parkinson's Disease Questionnaire
CMP: Chinese medicine practitioner
SPIRIT: Standard Protocol Items: Recommendations for Interventional Trials
HKBU: Hong Kong Baptist University
CUHK: The Faculty of Medicine of The Chinese University of Hong Kong
QEH: The Queen Elizabeth Hospital
RA-I: Research assistant I
HK-MOCA 5 min-protocol: The Hong Kong version of the Montreal Cognitive Assessment 5-min protocol
RA-II: Research assistant II
GMP: Good Manufacturing Practice
MDS-UPDRS: Movement Disorder Society Sponsored Revision of Unified Parkinson’s Disease Rating Scale
NMSS: Non-motor symptom scale
CTCAE: Common Terminology Criteria for Adverse Events
PI: Principal Investigator
RA-III: Research assistant III
