# Supplementary material for: Chinese herbal medicine treatment based on subgroup differentiation as adjunct therapy for Parkinson’s disease: study protocol of a pilot add-on, randomised, controlled, pragmatic clinical trial
Source: Chin Med. 2022 Jan 24;17:16. doi: 10.1186/s13020-022-00572-0 (PMC8785505; doi:10.1186/s13020-022-00572-0)
Supplement: Supplementary file 2 — Additional file 2. World Health Organization Trial Registration Data Set (this document outlines the basic information of this trial as registered on the clinical trial registry). [file 13020_2022_572_MOESM2_ESM.pdf]

World Health Organization Trial Registration Data Set	
**Data category**	**Information**
Primary registry and trial identifying number	ClinicalTrials.gov<br>NCT05001217
Date of registration in primary registry	10 October, 2021
Secondary identifying numbers	20B2/002A, REC/20-21/0206
Source(s) of monetary or material support	Chinese Medicine Development Fund
Primary sponsor	Hong Kong Baptist University
Secondary sponsor(s)	Not applicable
Contact for public and scientific queries	Professor Min Li, MD, PhD<br><br>Tel: (+852) 34112919<br><br>Email: limin@hkbu.edu.hk
Public title	Chinese herbal medicine treatment based on subgroup differentiation as adjunct therapy for Parkinson’s disease
Scientific title	Chinese herbal medicine treatment based on subgroup differentiation as adjunct therapy for Parkinson’s disease: study protocol of a pilot add-on, randomised, controlled, pragmatic clinical trial
Countries of recruitment	Hong Kong

**World Health Organization Trial Registration Data Set**	
**Data category**	**Information**
Health condition(s) or problem(s) studied	Parkinson’s disease
Intervention(s)	Active comparator: conventional antiparkinsonian medications, including levodopa, dopamine agonist, Monoamine oxidase-B, Catechol O-methyltransferase, Antimuscarinics and Amantadine<br><br>Experimental arm: Chinese herbal medicine based on a clinical guideline plus conventional medications
Key inclusion and exclusion criteria	Inclusion criteria:<br><br>- diagnosed with PD according to United Kingdom Parkinson's Disease Society Brain Bank (UKPDBB) diagnostic criteria<br>- aged 18 to 80 years old<br>- under stable ConM treatment with no alteration of dosage in the past 30 days<br><br>Exclusion criteria:<br><br>- secondary PD or atypical parkinsonian disorder<br>- used antidepressants in the past 30 days<br>- suffering from psychiatric, mood, or other neurological disorders<br>- suicidal (with suicidal thoughts in the past year)<br>- history of severe diseases, such as cancer and myocardial infarction<br>- participation in another clinical study<br>- pregnant or breast-feeding; (8) Hoehn and Yahr (H&Y) stage 4 or above<br>- liver and renal function derangement<br>- does not speak Chinese or English<br>- Hong Kong version of Montreal Cognitive Assessment 5-minute protocol < 22 points (the cut off for patients susceptible to mild neurocognitive disorder).

World Health Organization Trial Registration Data Set	
**Data category**	**Information**
Study type	Interventional<br>Allocation: randomized<br>Intervention model: parallel assignment<br>Masking: open-label (assessor and data-analyser blinded)<br>Primary purpose: treatment<br>Phase III
Date of first enrolment	Dec 2021
Target sample size	160
Recruitment status	Recruiting
Primary outcome(s)	the Movement Disorder Society Sponsored Revision of Unified Parkinson's Disease Rating Scale (MDS-UPDRS) Part II
Key secondary outcomes	sub-total scores in Part I, III and IV as well as the total score of MDS-UPDRS, and the domain and total score of Non-motor symptom scale (NMSS)
